# New Personalized Medicine Model for Medication Management

**DOI:** 10.3390/jpm16040182

**Published:** 2026-03-27

**Authors:** Kannayiram Alagiakrishnan, Tyler Halverson, Desiree Virginia Fermin Olivares, Cheryl A. Sadowski

**Affiliations:** 1 Division of Geriatric Medicine, University of Alberta, 11350-83 Ave, Edmonton, AB T6G 2P4, Canada; 2 Clinical Sciences Building, University of Alberta Hospital, 11350-83 Ave, Edmonton, AB T6G 2P4, Canada; 3 Child and Adolescent Mental Health Services, Trillium Health Partners—Credit Valley Hospital, Mississauga, ON L5M 2N1, Canada; 4 College of Pharmacy, University of Michigan, Ann Arbor, MI 48109, USA; fdesiree@umich.edu; 5 Faculty of Pharmacy & Pharmaceutical Sciences, University of Alberta, Edmonton, AB T6G 2H1, Canada; cherylas@ualberta.ca

**Keywords:** pharmacokinetics, pharmacodynamics, pharmacomicrobiomics, microbiome, pharmacomulti-omics, pharmacogenetics, computational biology

## Abstract

When using traditional approaches, such as pharmacokinetics and pharmacodynamics, the entire cellular or molecular response to drugs in the body cannot be fully ascertained or established. The oral medication process involves pharmacokinetics, followed by oral microbiomics and then gut microbiomics and pharmacodynamics. Recently, there has been increasing interest in the role of genetics (pharmacogenetics and pharmacogenomics) in both humans and microbiomes, as well as omics alterations (e.g., epigenetic, transcriptomic, proteomic, and metabolomic alterations as a consequence of drug exposure), which can help to ascertain the cellular responses to medications. Both the efficacy and toxicity of a drug are influenced by these factors. To assess these at an individual level, an integrative Personalized Medicine Model may be needed to help with medication management. Two example application cases for SSRIs and statins demonstrate the clinical usefulness of such a model, which can guide clinicians during drug selection and dosing to reduce reliance on trial-and-error, thus potentially improving patient outcomes and safety. Integrating this framework into practical clinical workflows requires the capture, analysis, and translation of multi-omics data in order to realize decision support protocols and actionable drug recommendations. This review also discusses IT requirements and different stakeholder roles. Although the proposed model can guide the treatment of diseases at the individual patient level, further research is still needed before it can be implemented as part of drug development research, clinical care, and healthcare delivery systems.

## 1. Introduction

Pharmacotherapy—referring to the use of drugs to treat diseases—is a key component in the management of most acute and chronic medical conditions. Traditionally, the two major areas of pharmaceutical research were pharmacokinetics and pharmacodynamics. Pharmacokinetics is the study of how the body handles medication with regard to absorption, distribution, metabolism, and excretion (ADME), whereas pharmacodynamics describes the therapeutic effects of the drug on the body and how well the target cells (for example, heart tissue cells or neurons) respond to the drug. The oral medication process involves pharmacokinetics, followed by oral microbiomics and then gut microbiomics and pharmacodynamics [1].

In clinical settings, the standard approach involves treating different individuals with the same medical condition with the same recommended starting dose of a particular medication. However, the individual differences between people with regard to genetics, comorbidities, and lifestyle may affect their health status and alter their response to the medication [2,3]. Biomarkers like metabolites and different proteins can help clinicians to not only monitoring disease progression, but also provide tailored treatments [4,5].

Due to individual differences, responses to pharmaceutical treatments vary and may be ineffective in 30–60% of patients [6]. Individual variability in drug response (IVDR) ranges from 50 to 75% [7], thus increasing the difficulty of predicting drug efficacy and identifying patients who are at risk for adverse drug reactions (ADRs). Therefore, using a standard treatment at the recommended dose may predispose some patients to an adverse drug reaction, with the incidence of fatal ADRs having been reported to be 0.32% [8]. Moreover, about 10% of hospitalized patients experience an ADR, and 3.5% of hospital admissions in Europe are related to ADRs [9]. Serious drug toxicities in the USA cause over 100,000 deaths and also cost billions annually [10]. Therefore, IVDR is responsible for not only poorer health-related outcomes, but also economic burdens.

Especially in the heterogenous group of older adults, a one-size-fits-all approach is not effective since all the characteristics that contribute to pathological processes—including genetics, epigenetics, multi-omics (e.g., proteomics and metabolomics), lifestyle factors, and nutrition intake—differ between individuals. Moreover, the enormous inter-patient diversity in human gut microbiomes and inter-related factors such as diet, circadian rhythms, and immune function are significant contributors to the variability in drug disposition and response [11].

Exposomics focuses on the interactions between environmental exposures and human health. It encompasses the comprehensive analysis of external factors such as pollutants, chemicals, lifestyle choices, and social determinants that influence health outcomes and disease susceptibility. By integrating data from various omics disciplines with environmental and lifestyle factors, exposomics offers valuable insights into disease etiology, risk assessment, and preventive strategies in precision/personalized medicine.

Traditional healthcare models struggle to address the complexity of modern healthcare needs, particularly given the increasingly multimodal nature of health data involving genetic, clinical, behavioral, environmental, and lifestyle information [12,13]. As precision/personalized medicine emerges as a promising solution for integrating multimodal data into healthcare, a new implementation strategy is necessary due to the complexity of existing healthcare structures [14].

Personalized medicine (PM) has emerged as a necessary approach for the prevention, diagnosis, and treatment of conditions [15]. Thus, PM is not considered a medical revolution, but an evolution [16]. In this review, we discuss a new model of traditional pharmacokinetic and pharmacodynamic frameworks that integrates pharmacogenomics, pharmacomicrobiomics, pharmacometabolomics, and multi-omics approaches, supported by computational biology and a possible new schematic framework with multimodal data integration to support clinical decision making.

## 2. Precision Medicine Versus Personalized Medicine: Why Is There a Need for a New Personalized Medicine Model?

Precision medicine is an approach that tailors medical treatment to individual characteristics such as lifestyle, environment, and genetics. By focusing on these individual variabilities, precision medicine aims to provide more effective and targeted treatments compared to the traditional “one-size-fits-all” approaches in conventional medicine, which are based on population averages. The terms “precision medicine” and “personalized medicine” are often used interchangeably, though some point out a subtle distinction. Nonetheless, these approaches represent distinct paradigms with unique methodologies and implications for patient care. A summary of the comparison between Conventional medicine and Precision/Personalized medicine. 

Summary of key distinctions between models.
**Feature****Conventional Medicine****Precision/Personalized Medicine****Treatment Model**“One-size-fits-all”Tailored to patient characteristics**Basis of Decisions**Population averages, symptomsIndividual biological and lifestyle data**Key Tools**Physical exam, general lab testsGenomic sequencing, biomarkers, AI/multimodal data**Goal**To treat the disease based on evidence from clinical trialsTo optimize treatment for a specific patient/subgroup Integration occurs at single omics level (genomics) (precision medicine) Integration at various multi-omics levels (personalized medicine)

While some use “personalized medicine” to refer to the broader concept of holistic care (including the patient’s social context and preferences), and “precision medicine” to refer to the data-driven, biological approach, both terms generally describe the modern movement away from conventional medicine. Overall, personalized medicine is a therapeutic approach that takes into account a patient’s genetic makeup, integrating their knowledge, environment, preferences, social context, and other daily life factors into a tailored treatment plan. Under the Personalized Medicine Model, the genetic basis of disease, combined with individual characteristics (e.g., based on molecular profiling, lifestyle data, multi-omics with data integration at multiple levels, and patient involvement in the decision-making process), is used for the medication-based management of diseases [17].

Rather than tailor treatment plans to the patient, precision medicine is used to classify individuals into subpopulations that differ in their susceptibility to a particular disease based on the biology or prognosis of the diseases they may develop or on their response to a specific treatment. For instance, to predict variability in drug responses, it is important to consider not only genetic factors, but also environmental microbiome-related factors and multi-omics factors. Therefore, it is crucial to take everything into account when implementing a treatment plan at a clinic. Currently, some precision medicine inputs remain insufficient. Pharmacogenomics is a comprehensive approach used to understand the genetic basis of drug response involving multiple genes along with other factors like transcriptomics and proteomics (which reflect the genetic makeup of the individual). However, pharmacogenomics can only explain a limited proportion of IVDR. The gut microbiome has a second genome and its metabolites can affect medication-related outcomes. Hence, pharmacomicrobiomics and pharmacometabolomics should also be considered.

In personalized medicine, different criteria, including diagnostic testing, are used to select medications based on the patient’s cellular or molecular characteristics. Thus, there is a need to integrate all of these approaches, which can be realized under the proposed Personalized Medicine Model.

While existing precision medicine approaches do not consider (detailed) multi-omics information and integrated pipelines streamline workflows, integrated multi-omics pipelines translate high-dimensional biological data into actionable knowledge by enabling the simultaneous investigation of different molecular layers and providing a holistic view of biological systems. These advanced pipelines also improve the reproducibility and accessibility of next-generation sequencing analyses in order to overcome the limitations of precision medicine. With this in mind, in this review, we propose a new model for personalized medicine, which we discuss in detail below, and consider the application of this model to two clinical scenarios. This review also proposes an integrated pharmacological and implementation framework, bridging the capture of multi-omics data with clinical workflows to translate molecular data into actionable, decision-supported drug recommendations supported by robust IT infrastructure and stakeholder roles.

Multi-omics integration pipelines—such as MOFA (Multi-Omics Factor Analysis), DIABLO (Data Integration Analysis for Biomarker Discovery using Latent Variable Approaches for Omics Studies), and Similarity Network Fusion (SNF)—are technical workflows designed to combine, normalize, and interpret heterogeneous datasets (genomics, transcriptomics, proteomics, and metabolomics) to understand complex biological systems. Multi-omics integration pipelines are distinct from, but enable, technical foundations for personalized medicine; that is, while personalized medicine is the clinical goal, multi-omics pipelines are the computational tools (software and algorithms) used to analyze data at different molecular levels—such as genomic, transcriptomic, proteomic, and metabolomic data—to reach this goal. Personalized medicine is a healthcare strategy that utilizes these integrated data, along with environmental and lifestyle factors, to provide targeted treatment.

Machine learning genome-based precision medicine models do not include microbiome data. Conversely, we integrate pharmacomicrobiomics into our proposed model. The machine learning-based approach lacks interdisciplinary collaboration between healthcare providers as well as validation to fully enable clinical decisions; in other words, it lacks clinical applicability.

Our proposed Personalized Medicine Model requires new equations to assess potency/efficacy trade-offs.

To properly validate a personalized medicine intervention across pilot, cohort, and randomized control trial (RCT) phases, the analysis must move beyond general efficacy to specific, actionable, and patient-centered metrics.

For personalized medicine, success is defined not only by statistical superiority but also by how effectively the tool identifies the right treatment for the right person while reducing the burden on clinicians (reducing alert fatigue) and improving access for all patients (equity).

The proposed integrative framework in personalized medicine shifts the paradigm from reactive, single-omic, disease-focused stratification (typical of current precision medicine) to a proactive, multivariable, “whole-person” approach.

## 3. Different Steps in the Current Pharmacological Process (Please See Table 1 and Figure 1)

- Organ/systems mechanisms:
  Pharmacokinetics (PK);
  Pharmacodynamics (PD);
  Polypharmacy with pharmacokinetic and pharmacodynamic drug interactions.
- Molecular and cellular discrete mechanisms (viewed individually at this point):
  Pharmacogenomics;
  Pharmacomicrobiomics;
  Pharmacometabolomics;
  Pharmacomulti-omics.
- Signal transduction/cellular communication (cell signaling) mechanisms:
  Receptor/post-receptor effects.

**Table 1:** Clinical and implementation endpoints for Personalized Medicine Model.

Clinical Endpoints	Implementation Endpoints
**Adverse Drug Reaction (ADR) Reduction Rate:** Reduction in pharmacy-reported or EHR-detected ADRs related to the drug when using personalized treatment compared with standard care.	**Alert Fatigue (CDS Burden):** Number of interruptive alerts fired per 100 medication orders and the ratio of active (hard-stop) vs. passive (informational) alerts.
**Time-to-Response/Optimal Dosage:** Number of days required to reach therapeutic range, symptom remission, or stable maintenance dosage compared with standard trial-and-error titration.	**Acceptance Rate:** Percentage of actionable clinical decision support (CDS) alerts that are accepted by clinicians (e.g., medication changed or dose modified).
**Hospitalizations/Readmissions:** 30-day hospital readmission rates or emergency department visits related to medication failure or toxicity.	**Override Rate and Reasons:** Frequency of clinicians bypassing CDS recommendations and the documented justification (e.g., “patient already stable,” “incorrect patient phenotype”).
**Treatment Discontinuation:** Percentage of patients who discontinue therapy due to side effects or lack of efficacy within a defined timeframe.	**Time Burden:** Average “think time” or “dwell time” between the appearance of a CDS alert and the clinician’s action.

### 3.1. Pharmacokinetics and Pharmacodynamics/Pharmacodynamic and Pharmacokinetic Drug Interactions

Pharmacokinetics is the study of how the body handles medication and explains the relationship between the dose and response. Pharmacodynamics describes what a drug does to the body.

Note that although traditional approaches like pharmacokinetics and pharmacodynamics do not allow the entire cellular or molecular response to drugs in the body to be fully ascertained or established, they are still essential components in clinical trials as they facilitate the development of new drugs.

Pharmacokinetic drug interactions occur when a drug alters the ADME of a drug that is administered or co-administered [1]. For instance, drugs that increase the stomach pH, such as antacids and proton pump inhibitors, can alter the kinetics of co-administered agents that require an acidic pH for dissolution and absorption [18]. Conversely, pharmacodynamic drug interactions occur when co-administered drugs act on the same receptor target. For example, buprenorphine is a partial μ-opioid receptor agonist with high affinity that can functionally antagonize the effects of full μ-opioid agonists, such as morphine [19].

A personalized medicine approach can help to elucidate drug–drug interactions better, not only in terms of shared metabolic pathways but also with regard to drug–microbiome and drug–metabolite interactions.

### 3.2. Pharmacogenetics and Pharmacogenomics

Pharmacogenetics is the study of how a single gene affects the variation in an individual’s drug response, while pharmacogenomics is the general case of pharmacogenetics, which reflects how diversity in multiple genes affects the response to a single drug. Progress has been made in determining genetic variability in drug-metabolizing enzymes, drug transporters, and drug target genes, resulting in clinically actionable guidelines for selecting drugs [20]. Modern approaches focus on translating genetic data into immediately actionable, patient-specific, and digitized insights that directly guide prescribing decisions.

Pharmacogenetics may account for 20–95% of the individual variability in drug responses [21,22,23,24,25,26,27,28,29,30]. Pharmacogenomic approaches are associated with different treatment responses, remission, resistance, and adverse drug reactions [31].

**Figure 1:**
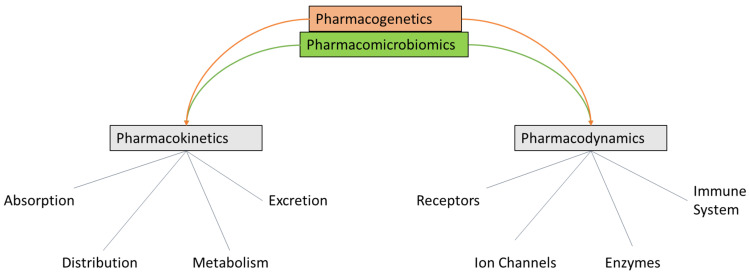
Current model of the roles of pharmacokinetics and pharmacodynamics in drug–host interactions. Our current understanding incorporates the roles of pharmacogenetics and pharmacomicrobiomics in drug reactions experienced by individuals. The figure was adapted and modified from Pirmohamed and Park (2001) [31].

Pharmacoepigenetics refers to the behavioral and environmental factors that cause epigenetic modifications and alter gene expression, thereby influencing an individual’s drug response. With aging, epigenetic alterations occur, affecting pharmacokinetics (through CYPs (cytochrome p450) and ABCs (ATPase-binding cassette and P-glycoproteins)). Regarding changes in pharmacodynamics, epigenetic alterations can affect the expression, availability, and overall function of common receptors; for example, alterations to the function of monoaminergic receptors and decreased permissive and increased repressive histone markers at important gene promoters are known to be (at least partially) responsible for the increased side effects and decreased efficacy of drugs. Moreover, studies on antipsychotics have found that epigenetic alterations can affect the expression of specific enzymes and transporters. This can lead to decreased clearance and changes in the distribution of antipsychotics in the central nervous systems (CNS) [32]. Pharmacological processes and their definitions are highlighted in Table 2, which serve as the basis for the current model of pharmacokinetics and pharmacodynamics, but are also important elements within the personalized medicine framework.

Pharmacoepigenetic therapy involves developing drugs that target aberrant epigenetic patterns to activate or suppress gene expression. Epigenetic modifications—including DNA methylation, histone modification, covalent modifications, non-coding RNAs, and nucleosome positioning—affect an individual’s response to drugs by influencing drug transporters, metabolic enzymes, and nuclear receptors. This is known as pharmacoepigenetic therapy, which targets aberrant epigenetic patterns that either activate or suppress the epigenetically modified genes’ expression [23,24,25,26,27,28,29,30,31,32,35,42,43]. For example, SGLT-2 inhibitors can influence DNA methylation, histone modifications, and miRNA regulation and contribute to beneficial effects on the heart [44]. Metformin can modify histones by influencing the activities of enzymes involved in DNA methylation, which contributes to its potential anti-cancer benefits [45,46].

The distinction between genetics and epigenetics is that genetic processes are irreversible, whereas epigenetic processes can be reversible [47]. These alterations can result in a variety of diseases, such as cancer, schizophrenia, neurodegenerative diseases, and autoimmune disorders. They can be passed down from the daughter cells through the splitting process [48]. Epigenetic alterations come in a variety of forms, including DNA methylation, histone modifications, chromatin remodeling, and microRNAs, all of which have regulatory functions [49] and are involved in the regulation of genes and a number of biological functions related to a number of diseases [50].

Epigenetic therapies are being developed for various diseases, including cancer, cardiovascular disease, neurological disorders, and metabolic diseases, which target and modify gene expression without altering the underlying DNA sequence. These therapies involve the use of drugs to influence epigenetic mechanisms like DNA methylation and histone modification to correct aberrant gene activity that contributes to diseases.

In personalized medicine, epigenetic therapies hold promise for creating more personalized treatment approaches based on an individual’s unique epigenetic profile.

### 3.3. Pharmacotranscriptomics

Pharmacotranscriptomics refers to the comprehensive study of all RNA molecules within a cell or tissue in the context of pharmaceutical research and practice.

Regarding integration with other omics, transcriptomics has been integrated with genomics, proteomics, and metabolomics to provide a holistic understanding of disease mechanisms and drug actions, as gene expression levels do not always perfectly correlate with protein abundance or activity [51].

### 3.4. Pharmacomicrobiomics

The gut microbiota includes trillions of microorganisms, such as bacteria, archaea, fungi, and viruses, that can influence human health and diseases by interacting both directly with drugs and indirectly through their effects on the host, who, in turn, exerts an effect and influence on medications [52].

Rizkallah et al. coined the term pharmacomicrobiomics in 2010 [36], which refers to the study of interactions between the gut microbiota, the host, and medications. Microbial interactions are bidirectional as the microbiome and drugs can affect each other. The gut microbiome can directly metabolize orally administered drugs into secondary products. Drug bioavailability depends on microbial modification. Microbial metabolites such as bile acids also have an effect on drug disposition. The intestinal bacterial flora produce secondary bile acids by metabolizing primary bile acids synthesized by the liver. Through processes like deconjugation and 7-dehydroxylation, bacteria (e.g., *Clostridium* and *Lactobacillus*) convert primary bile acids into various secondary forms that regulate digestion, drug metabolism, and immune function [53].

Microbes and their products also play a role in the gut–liver crosstalk; they have an effect on enterohepatic recycling, intestinal and hepatic drug metabolism, and transporters affecting total body drug exposure [54].

Pharmacogenomics and pharmacomicrobiomics are related fields involving the study of how an individual’s biology affects their response to medications. In particular, pharmacogenomics studies genetic variations, such as single-nucleotide polymorphisms (SNPs) in enzyme systems like CYP450, helping us to identify patients who may experience drug side effects. Meanwhile, pharmacomicrobiomics estimates the effects of the human microbiome and environmental factors on human pharmacokinetics (PK) and pharmacodynamics (PD), as well as gene expression related to metabolism.

Pharmacomicrobiomics plays a role in the variability in drug responses and may play a further role in adverse reactions. Genetics (host and microbiome genetics), comorbid diseases, environmental factors, and diet are factors that play a role in pharmacomicrobiomics.

Studies in animals and humans have shown that changes in the gut bacteria can also occur due to diseases such as diabetes, heart disease, cancer, and certain neurological diseases [55,56,57,58,59,60,61,62,63,64,65]. Medications like proton pump inhibitors (PPIs,) metformin, laxatives, statins, antidepressants, and opioids can explain some of the variability in gut microbiome composition in these studies [65,66,67,68]. The Dutch LifeLines-DEEP cohort [69] has shown changes in gut bacteria due to antibiotics and other microbiome-associated drugs, including PPIs, lipid-lowering statins, laxatives, metformin, beta-blockers, ACE inhibitors, and selective serotonin reuptake inhibitors. Human cohort-based analyses have further revealed that the dynamic nature of the gut ecosystem reflects complex interactions between the host and lifestyle, dietary, ecological, and other factors (e.g., medications, disease, and smoking) [69,70,71].

Gut microbial enzymes influence the bioavailability of oral drugs by affecting the first-pass effect of enterohepatic circulation as well as their metabolism [72]. Gut microbiome biotransformation can also inactivate drugs; for example, in around 10% of patients, digoxin is converted to cardio-inactive reduction products [73]. Therefore, considering the contribution of the gut microbiome to drug disposition, drug concentration, pharmacokinetics, and pharmacodynamics is vital in the era of personalized medicine. Bioavailability is a very important factor to consider for the therapeutic efficacy of oral drugs [72]. Microbial products, such as bile acids, interact with nuclear receptors on host drug-metabolizing enzyme machinery, thus indirectly influencing drug disposition [54].

Recent estimates indicate the presence of 232 million gene-encoding organisms in the human gut microbiome [54], far outnumbering human germline genes (~20,000) [74]. More than 90% of the gut microbiota are members of two bacterial phyla, Bacteroidetes and Firmicutes [75], and the Firmicutes/Bacteroidetes ratio is used as an indicator of gut health [76].

As with other factors influencing individual variability, accounting for the influence of the microbiome is even more critical for drugs with a narrow therapeutic range. Currently, although the available data on the gut microbiome’s influence on drug pharmacokinetics and pharmacodynamics are not robust enough to translate into clinically actionable guidance in practice [77,78,79,80,81,82], they play an important role in drug development and clinical practice.

In addition to genetics, diet and other drugs (polypharmacy) can play a role in determining drug doses and responses. Thus, studying this highly variable and complex system will require a multi-pronged approach, leveraging animal, human, and systems biology models [82].

Clear evidence indicates that orally administered pharmaceutical excipients directly interact with gut microbes and can either positively or negatively impact the gut microbiota’s diversity and composition. However, these relationships and mechanisms have not been consistently investigated during drug formulation, despite the potential for excipient–microbiota interactions to alter drug pharmacokinetics and interfere with host metabolic health [83]. Despite clear evidence that inactive ingredients (e.g., binders, sweeteners, and surfactants) can alter drug pharmacokinetics and potentially cause adverse effects, these interactions are also rarely evaluated during drug formulation. Excipients can positively or negatively affect gut microbial communities, acting as prebiotics or causing dysbiosis. These interactions can alter the efficacy of the drug itself and affect host health, particularly during long-term use. Acknowledging these interactions is crucial for personalized medicine and reducing interindividual variability in drug response.

A profiling method that can distinguish between different microbial characteristics that may impact drug response is essential for pharmacomicrobiomics. Current techniques include shotgun metagenomic sequencing for functional and strain-level characterization [84] and 16S rRNA gene and Internal Transcribed Spacer (ITS) sequencing for taxonomic profiling of bacteria and fungi, respectively. In contrast, multi-omics techniques like metabolomics, metaproteomics, and metatranscriptomics offer a more comprehensive picture of the active microbial pathways and host–microbe interactions. These techniques have improved the detection of biomarkers for cancer and other illnesses [85]. Notably, multi-omics-based profiling can be used to identify disease-specific signatures, as opposed to solely looking at the microbiome composition [86].

A structured, multi-stage approach will be necessary to convert these results into clinical recommendations. In order to identify potential microbial genes, enzymes, and/or metabolites involved in medication metabolism [87], it is necessary to conduct several large-scale studies. After potential candidates have been found, they need to be validated in experimental models and eventually included into predictive computational frameworks, such as physiologically based pharmacokinetic (PBPK) modeling and machine learning classifiers [86]. Once validated microbiome-derived biomarkers have been identified, they should be evaluated in clinical groups and, preferably, tested in randomized controlled studies before being integrated into practical recommendations [86]. To enhance replication across studies and laboratories, it will be necessary to employ precise standardizing and reporting procedures for sample collection, processing, and data analysis [88]. The STORMS Microbiome Reporting Checklist [89] can be used to support this process. To bridge the current knowledge gap, future efforts must prioritize clinician training and knowledge transfer from researchers to clinicians. This will help to translate complex microbiome data into actionable, accurate, and evidence-based clinical guidelines.

It has been assumed that pharmacogenetics plays a crucial role in shedding light on variations in drug response among individuals and communities. It has been frequently employed to offer data on possible hazards related to negative medication responses, drug effectiveness, and the best drug dosage. Pharmacomicrobiomics can further build upon this, providing insight into the degree of variability produced by the microbiome in the interactions between the host and the drug. The microbiome is a dynamic and modifiable system with fluctuating composition and metabolic activity [90], whereas the human genome is a more static component of the host. As a result, these elements have the potential to impact drug disposition via microbial biotransformation, microbial-induced modulation of host drug-metabolizing enzymes, substrate competition, ecological changes in the gut microbiome, and alterations in the intestinal barrier [91,92]. Hence, by increasing our understanding of the interactions between microbes and drugs, we may gain better insight into personalized medicine.

#### Microbial Transformation

It is typically understood that there is a level of biotransformation with drug metabolism; however, this biotransformation can also occur through the actions of the gut microbiome. Microbial transformation occurs when drugs or medications are enzymatically modified by the host’s gut microbes; it may occur before intestinal absorption or within specific microbial niches, and can generate metabolites that substantially differ from those produced by host hepatic or intestinal enzymes. A well-established example is the microbial inactivation of digoxin by Eggerthella lenta, mediated by the cardiac glycoside reductase (cgr) operon [93]. Explicitly distinguishing microbial from host biotransformation allows for a better understanding of the interacting metabolic pathways that jointly shape drug exposure and therapeutic outcomes [92]. Along with microbial biotransformation, emerging research has highlighted the role of bioaccumulation within microbial cells/components [81]. It is thought that this microbial bioaccumulation contributes to the variation seen between individuals and populations in the pharmacokinetics of various drugs. It has been observed that certain pharmaceuticals, such as psychotropics, accumulate within specific gut bacterial taxa without undergoing any modifications or processing, which can impact luminal drug availability and systemic absorption [81]. Recognizing microbial bioaccumulation as part of the pharmacomicrobiomics framework helps to highlight the importance of microbe–drug interactions and their influence on drug disposition, not only through metabolic transformation but also through physical partitioning within microbial or host areas [94].

### 3.5. Pharmacometabolomics

Metabolomics is defined as “the measurement of metabolite concentrations and fluxes, and secretion in cells and tissues in which there is a direct connection between the genetic activity, protein activity, and the metabolic activity itself” [95]. Pharmacometabolomics is a subclass of metabolomics that studies how drugs affect an individual’s metabolism (and vice versa) by analyzing the body’s metabolites. It aims to evaluate the levels of small-molecule metabolites in bodily fluids such as plasma and/or urine, which may help in determining the efficacy and toxicity of drugs, with the aim of understanding drug responses, predicting their efficacy, and identifying potential adverse effects, leading to personalized medicine and more effective therapies [96]. Analytical techniques such as nuclear magnetic resonance (NMR), liquid chromatography–mass spectrometry (LC-MS), and gas chromatography–mass spectrometry (GC–MS) have been employed to assess a huge number of metabolites present in biological systems [97]. For instance, a metabolomic study on aspirin found differences in the levels of metabolites, specifically purine metabolites, between poor and good responders to the drug after a 2-week intervention [98].

Pharmacometabolomics focuses on the interplay between drugs and the body’s metabolism, aiming to improve patient safety and personalize healthcare. While pharmacogenomics informs us of what might happen, pharmacometabolomics (metabolism) tells us what is happening, capturing the influence of diet, gut bacteria, and the environment. In a practical sense, it can help clinicians to predict whether a treatment will work or result in toxic side effects [99].

With potent applications in drug development and discovery, metabolomics and pharmacometabolomics are at the forefront of personalized medicine. Currently, critical analytical approaches are being investigated to increase metabolome coverage, boost analytical efficiency, and lower costs; such methods include mass spectrometry and nuclear magnetic resonance spectroscopy, as well as analytical separation methods like liquid and gas chromatography, ion mobility spectrometry, capillary electrophoresis, and supercritical fluid chromatography. Major issues in metabolomics, such as metabolome complexity, data analysis and integration, and biomarker validation, are being addressed through continuous improvements in computational tools and instrumentation [100].

The combination of different analytical platforms allows for more comprehensive measurements of the entire metabolome, as no single technique can detect all metabolites.

### 3.6. Pharmacomulti-Omics

#### 3.6.1. Role of Multi-Omics in Personalized Medicine

This field is focused on the integration of different processes, encompassing genomics, proteomics, metabolomics, transcriptomics, epigenomics, and microbiomics. Advanced computational technologies such as artificial intelligence (AI) and machine learning (ML) play crucial roles in processing and integrating complex omics data, enhancing predictive accuracy and creating tailored therapeutic strategies, with the aim of moving away from “one-size-fits-all” approaches toward a more precise and patient-centered model of care [21,27,101,102,103,104,105].

Human microbiomes may produce some compounds that have an influence on drug pharmacokinetics and pharmacodynamics due to altered disease states; altered expression of metabolic enzymes, drug transporters, or genes coding for drug target proteins; or drug response phenotypes [106].

Early studies and small trials suggest that combining different types of data, known as multi-omics integration, could lead to more accurate and personalized treatments; however, this area is still developing and is not yet sufficiently established for regular clinical use.

Current evidence supporting multi-omics mostly comes from discovery studies that use data to improve disease diagnosis and identify new treatment targets (Table 3). Most findings are from data analysis and small clinical trials rather than large, thorough studies.

Strong evidence is needed to make multi-omics a recognized practice. This includes validating results in large, independent patient groups and conducting large observational studies to determine how well multi-omics predictions work in real-life situations. Critical missing evidence would come from randomized controlled trials (RCTs) that compare multi-omics treatments with standard care, demonstrating meaningful health improvements. Additionally, data showing that multi-omics approaches are cost-effective and can fit into current healthcare systems are also necessary for implementation. Overall, while current findings are promising, more high-level clinical trial data are needed before integration into clinical guidelines [107]. Du et al. (2025) showed that adding omics data significantly improved risk prediction for all 17 considered diseases when compared to models with clinical predictors alone [108].

**Table 3:** (**Part A**) Representative studies supporting composite omics approaches. (**Part B**) Representative studies with a multi-omics focus.

Part A			
Type of Study	Sample Size	Key Findings and Impact	Reference
Genomics	487,409	There was a population-level pharmacogenetic variation, with nearly 24% of participants being prescribed a drug for which they were predicted to have an atypical response. The authors suggest there is a need to consider the variants relevant to the pharmacogene of interest and the ancestral background of the patient when selecting a genotyping modality.	McInnes et al. 2020 [109]
Genomics	565,390	The Taiwan Precision Medicine Initiative (TPMI) used whole-genome sequencing, genotyping arrays, and EHR-linked clinical phenotype to link DNA data with longitudinal EMRs to support GWAS, PheWAS, PRS development, and pharmacogenomics. It addresses the under-representation of Han Chinese in genomic research, enables ancestry-specific risk prediction, and provides a scalable model for precision medicine.	Yang et al. 2025 [110]
Genomics		The Clinical Pharmacogenetics Implementation Consortium has published 23 guidelines (of which, 11 have been updated) covering 19 genes and 46 drugs across several therapeutic areas. The CPIC also now provides additional resources to facilitate the integration of pharmacogenetics into routine clinical practice, as well as electronic health records.	Relling et al. 2015 [111]
Microbiomics	Cohort: N = 99,556	The authors report extensive novel associations between the host microbiome and common metabolic disorders. The results provide new insight into associations between the gut microbiota and metabolic disorders to support the translation of gut microbiome findings into potential clinical practice.	Qu et al. 2025 [112]
Multi-Omics	Cohort N = 185 UC: N = 77 CD: N = 108	This study profiled baseline stool and blood from patients with moderate-to-severe CD or UC initiating anti-cytokine therapy (anti-TNF or -IL12/23) or anti-integrin therapy. Baseline microbial richness indicated preferential responses to anti-cytokine therapy and correlated with abundance of microbial species capable of 7α/β-dehydroxylation of primary to secondary bile acids. Serum signatures of immune-proteins reflecting microbial diversity identified patients more likely to achieve remission with anti-cytokine therapy.	Lee et al. 2021 [113]
MedDMS/Informatics		LogiqSuite supports precision medicine data management across patient care, research, and analytics. It has been applied in seven projects (oncology, cardiovascular, pulmonology, prehospital triage), enabling multi-center data integration, real-time analytics, machine learning, CDS model development, and compliance with international privacy regulations.	Jacobs et al. 2025 [114]
Proteomics	Middle aged to elderly adults: N = 3796 (proteome serum samples N = 7565)	This study examined 86 aging-related proteins that exhibit signatures associated with 32 clinical traits and the incidence of 14 major aging-related chronic diseases. The authors obtained insight into the roles of serum proteins in aging and age-associated cardiometabolic diseases and provide potential targets for intervention with therapeutics to promote healthy aging.	Tang et al. 2025 [115]
**Part B**			
**Muti-Omics Layer(s)**	**Cohort/Sample Size**	**Outcome/Findings**	**Reference**
Metabolomics Metagenomics Metatranscriptomics Microbiomics Proteomics	N = 106 healthy individuals and individuals with prediabetes Collected over approximately 4 years	It was determined that healthy profiles are distinct among individuals while displaying diverse patterns of intra- and/or inter-personal variability. It was found that specific host–microbe interactions differ between insulin-resistant and insulin-sensitive individuals. This study provided an open access data resource to enable further research into healthy, prediabetic, and T2DM states.	Zhou W. et al. (2019) [62]
Host transcriptomics Metabolomics Metagenomics Metatranscriptomics Proteomics	N = 132 subjects for one year Longitudinal molecular profiles were generated for host and microbial activity during disease course	The study found a characteristic increase in facultative anaerobes, at the expense of obligate anaerobes, and molecular disruptions in microbial transcription, metabolite pools, and levels of antibodies in host serum. It also found that periods of disease activity were also marked by increases in temporal variability, with characteristic taxonomic, functional, and biochemical shifts.	Lloyd-Price J. et al. (2019) [64]
Population Metagenomics Deep Phenotyping	Belgian Flemish Gut Flora Project discovery cohort N = 1106 Dutch LifeLines-DEEP study (replication) N = 1135 Integration with global datasets Combined N = 3948	In this study, although the core microbiota from 14 genera was found, 664 identified genera remain underexplored with regard to total gut diversity. Sixty-nine clinical and questionnaire-based covariates were found to be associated with the microbiota compositional variation, with a 92% replication rate.	Falony G et al. (2016) [70]
Metabolomics Microbiomics + Host Markers	Cohort N = 185 UC: N = 77 CD: N = 108 Baseline stool and blood samples in patients with moderate-to-severe CD or UC were profiled at the initiation of anti-cytokine therapy (anti-TNF or -IL12/23) or anti-integrin therapy	Baseline microbial richness indicated preferential responses to anti-cytokine therapy and correlated with the abundance of microbial species capable of 7α/β-dehydroxylation of primary to secondary bile acids. Serum signatures of immune proteins reflecting microbial diversity identified patients more likely to achieve remission with anti-cytokine therapy.	Lee et al. (2021) [113]
Genomics Proteomics Transcriptomics	The Pan-Cancer Atlas initiative involved over 10,000 samples, covering 33 different cancer types	The project identified 299 cancer-driver genes and over 3400 driver mutations, which highlighted the molecular foundations of cancer. This allowed for the classification of distinct solid tumors based on their molecular profiles. This has led to the development of targeted therapies that have received approval for certain cancer types.	Weinstein et al. 2013 [116]
Genomics Metabolomics Transcriptomics	The gut microbiota of 531 Finnish men from the METSIM study was profiled using 16S rRNA gene sequencing	The study was able to identify novel associations between gut microbiota and fasting serum levels of a number of metabolites, including fatty acids, amino acids, lipids, and glucose. Notably, associations of fasting plasma TMAO levels with CAD and stroke were reported.	Org et al. (2017) [117]

Crohn’s Disease (CD), coronary artery disease (CAD), Interleukin (IL), Metabolic Syndrome in Men (METSIM), Tumor Necrosis Factor (TNF), Trimethylamine N-oxide (TMAO), Type 2 Diabetes Mellitus (T2DM), Ulcerative Colitis (UC).

**The key omics layers include the following: **

- Genomics: DNA sequences (potential disease predispositions);
- Transcriptomics: RNA expression (active genes);
- Proteomics: Proteins (functional machinery);
- Metabolomics: Metabolites (final products of biological processes);
- Epigenomics: Modifications to DNA that affect gene activity.

#### 3.6.2. Multi-Omics: A Symphony of Biological Data

When integrated, these individual omics data form what we call multi-omics. This approach allows for the capture of a complex interplay between different biological levels, providing a more holistic view of health and disease. It could be compared to listening to the performance of a full orchestra, where the individual instruments (the “omics”) come together to create a symphony of biological data.

Integrated multi-omics is the combination of multiple omics data layered over each other, including the interconnections and interactions between them, which helps us to understand human health and disease better than when considering any of the data separately [118,119]. By integrating omics, good molecular biology information flow is achieved, starting with DNA (genomics/epigenomics), which is transcribed into RNA (transcriptomics) and translated into proteins (proteomics) to facilitate metabolic reactions (metabolomics). This combined multi-omics analysis offers a more complete understanding of disease mechanisms, enabling precise diagnostics and personalized treatment. These layers respond to changes in the environment and disease state, allowing for the mapping of complex biological pathways, not just static genetic traits [120].

Phenomenal advancements in bioinformatics, data sciences, and artificial intelligence have made integrative multi-omics feasible, helping us to understand human health and disease better.

### 3.7. Molecular and Cellular Mechanisms, Including Signal Transduction/Cellular Communication (Cell Signaling), Signaling Pathways, and Signal Transduction Mechanisms

Cell signaling is a communication system between cells. It can occur over short or long distances and through different signaling pathways, which include endocrine (long-range communication), paracrine (short-range/localized), juxtracrine (contact-dependent signaling), autocrine (acting on the same cell that produces the factor), and neuronal neurotransmitter-mediated (signaling at synaptic junctions) signaling. Signaling pathways are responsible for converting extracellular signals into intracellular responses, thereby regulating cell function, and disturbances of these pathways are often seen in diseases. Essentially, drugs can target these disrupted pathways in order to produce more effective and targeted therapeutic effects for diseases involving cell signaling mechanisms or disruptions [41,121]. A signal transduction pathway is a series of molecular interactions triggered by the binding of a signaling molecule to its receptor, leading to the activation of various intracellular pathways involved in cell signaling. Signals or ligands perturb cellular homeostasis due to mechanical (mechanotransduction), electrical (electrotransduction), or chemical (chemotransduction) stimulation [122].

The New Personalized Medicine Model has two components:

1. The foundation—PK and PD;
2. The architecture—foundation + pharmacogenetics + pharmacomicrobiomics + pharmacometabolomics + multi-omics pharmacology + computational biology + multimodal data analysis.

#### 3.7.1. Integration Across Domains in the Personalized Medicine Model

The model must include basic labs, a medication list, and the genetic profile (multi-omics data). Furthermore, the following steps must be considered:

How are inputs weighted/combined?

Inputs are weighted based on the strength of evidence; for example, pharmacogenomic data for statin is weighted more than pharmacomicrobiomic data due to the strength of evidence.

How is uncertainty handled (signal vs. noise)?

As in the point above, the strength of evidence dictates certainty; the highest-level evidence prevails.

What is the output (risk class, ranked drug lists, dose ranges, and monitoring plans)?
Each input provides its own individual output. For example, consider the following:

Genomics—Phenotype metabolism data are provided for dose adjustments and monitoring.

Microbiome—The predicted efficacy/toxicity is derived from PK/PD and genomic data.

The model requires a core set of inputs to generate recommendations, including clinical data, medication history, basic labs, and actionable pharmacogenes (e.g., CYP2D6/CYP2C19 for SSRIs and SLCO1B1 for statins). Optional enhancements such as multi-omics, microbiome data, exposomics, and lifestyle/social factors refine predictions.

#### 3.7.2. How Inputs Are Weighted and Integrated

Data are combined using a tiered framework in which validated pharmacogenes, organ function, and high certainty PK/PD features carry the greatest weight, while metabolomic/microbiome findings serve as refiners rather than primary determinants. Integration uses multimodal fusion methods constrained by physiologic PK/PD principles to ensure clinically plausible outputs.

#### 3.7.3. Handling Uncertainty (Signal vs. Noise)

Uncertainty is managed through assay-level QC, probabilistic modeling, and confidence scoring, which classify outputs as high-, moderate-, or low-confidence. When uncertainty is high or data are missing, the system defaults to guideline-based dosing and monitoring to avoid the overinterpretation of weak signals.

#### 3.7.4. Model Outputs

The system produces four clear deliverables: (1) risk stratification for efficacy and adverse events, (2) a ranked medication list, (3) personalized dosing/titration suggestions, and (4) a tailored monitoring plan with pharmacist or genetic counselor involvement. Outputs include brief rationales and confidence levels to support rapid clinical decision making.

#### 3.7.5. Workflow Expansion

The workflow includes sample collection, data processing, bioinformatic analysis, clinical significance review, EMR integration, and pharmacist-supported recommendations. Actionable findings trigger CDS alerts and collaborative decision making, while non-actionable results are documented without interrupting the workflow.

**Figure 2:**
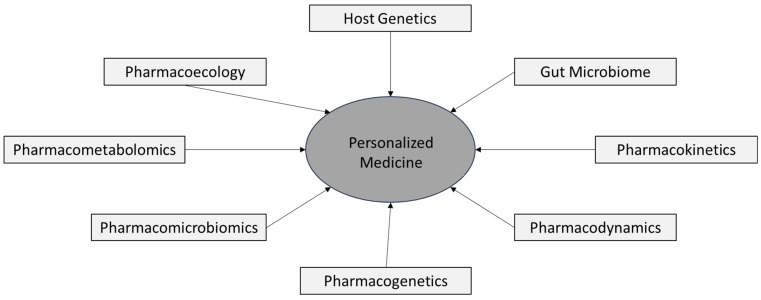
Factors that influence and contribute to personalized medicine. Various factors contribute to the Personalized Medicine Model through host, microbiome, and metabolic pathways. Within the host, there are specific “omic” domains, including genomics/pharmacogenetics, transcriptomics, proteomics, and metabolomics/pharmacometabolomics. There are also specific “omic” domains that are microbiome-related, including pharmacoecology and pharmacomicrobiomics. Pharmacokinetics and pharmacodynamics also play critical roles as they represent downstream functional outputs that integrate these upstream biological signals. The figure highlights how integrating these data streams enables a comprehensive, systems-level understanding of individual drug responses.

As shown in Table 4, compared to the conventional medicine model, the multi-omics Personalized Medicine Model varies in the features mentioned below. The entire outlook of personalized medicine is summarized in Figure 2. The proposed Personalized Medicine Model should integrate pharmacology to understand drug response pathways better than when considering the traditional model for predicting drug efficacy and toxicity. This should help in fully understanding the interactions of medications with underlying diseases and different biological systems through molecular mechanisms and interactions. Integrating pharmacology with molecular pathology—which examines molecules such as DNA, RNA, and proteins within tissues and organs—can provide new insights into how drugs interact with individual patients at the molecular level [123]. This model could help to advance personalized medicine by tailoring therapies based on individual genetic and molecular profiles [124].

The “multiple drugs → multiple targets → multiple diseases” model is a novel framework that recognizes that most medications interact with several targets, with broad consequences across various biological pathways. By integrating multi-omics data—such as genomic, transcriptomic, and proteomic data—into a biological network analysis framework, researchers can develop a powerful drug prediction method. This approach merges the detailed molecular profiling of multi-omics with the systemic perspective of network analysis, enabling more precise, mechanism-informed forecasts of drug efficacy, repurposing opportunities, and personalized medicine [125,126].

### 3.8. Computational Biology

Computational biology is an interdisciplinary field that uses computational methods to study biological systems and processes, combining computer science, mathematics, and data analysis to address complex biological questions [127]. Machine learning algorithms can analyze a large volume of data from different datasets of patient information (including electronic health records). With traditional guideline-based approaches, factors might be missed, even when identifying individuals at higher risk of medication side effects. With computational biology methods, one can improve medication efficacy and safety as well as tailor treatments to individual patients [128]. Computational biology can integrate genomic data, electronic health records, investigational records, and real-time patient monitoring information to identify disease patterns and recommend individualized treatment options as well as adaptive treatment plans tailored to dynamic patient needs.

Within computational science, artificial intelligence (AI), machine learning (ML), and deep learning (DL) form a hierarchical and synergistic framework, with each level reflecting different yet related levels of abstraction and capability. Theoretical modeling, simulation, and data-driven methodologies in computational chemistry have revolutionized the field of medicinal chemistry and made it a fundamental component of modern drug discovery [129]. The high expense, lengthy turnaround times, and high failure rates of past drug development have all been barriers. Computational chemistry can be employed to overcome these challenges by conducting in silico research on a compound’s physicochemical characteristics, molecular interactions, and pharmacokinetic profiles before it ever enters the lab phase [130,131]. The integration of artificial intelligence (AI) with computational chemistry has transformed drug development by improving compound optimization, predictive analytics, and molecular modeling [132].

#### 3.8.1. Data Harmonization

Data harmonization enhances data accuracy by combining, unifying, and standardizing data from various sources to assure accuracy, consistency, and usability. A common scenario involves a unified patient approach, where electronic health records (EHRs) from various providers are interfaced to hospitals to assure accurate diagnoses, efficient patient treatment, and adherence to Health Insurance Portability and Accountability Act (HIPAA) regulations, thereby encountering fewer administrative bottlenecks. Data harmonization is necessary for accurate analysis, efficient integration, and regulatory compliance. Standardizing and harmonizing different datasets can lead to the breakdown of data silos, increased interoperability, and improved decision making [133].

#### 3.8.2. Challenges in Data Harmonization

Metadata are crucial for understanding, sharing, and reusing datasets, but several challenges exist. First, terms can have different meanings across studies, leading to confusion. There is also a lack of standardized metadata schemas, making it difficult to compare datasets from different sources. Ensuring data quality requires careful human oversight and strict governance for accurate and updated metadata.

Interoperability—or the ability of different systems to work together—has its own issues. Different technical formats can hinder data transfer and, even when data are exchanged, systems may struggle to understand the information due to varying semantic standards. Older systems tend to not comply with current exchange protocols, thus creating data silos, and establishing ownership and trust between organizations can be difficult.

To address these challenges, several international projects and interoperability standards have been developed. The Health Level Seven International (HL7) Fast Healthcare Interoperability Resources (FHIR) standard enhances healthcare data exchange through modular and machine-readable “resources” that enable structured and interoperable sharing of clinical information [134]. The Global Alliance for Genomics and Health (GA4GH) provides technical standards and policy frameworks to enable responsible, secure, and interoperable genomic data sharing across international research and clinical environments [135]. The FAIR (Findable, Accessible, Interoperable, Reusable) guiding principles promote improved data stewardship and metadata practices to enhance interoperability and the reuse of scientific datasets [136]. Finally, common data models such as the Observational Medical Outcomes Partnership (OMOP) model standardize heterogeneous clinical datasets into a unified structure, enabling distributed research and multi-site analyses without requiring centralized data storage [137].

## 4. New Personalized Medicine Model for Two Common Medications Used in Clinical Practice

### 4.1. Example #1: SSRIs

With a lifetime prevalence of about 13% worldwide, major depressive disorder (MDD) is a prevalent mental illness [138,139]. A relative deficit in serotonin (5-HT) and other monoamine neurotransmitters in the central nervous system (CNS) play a significant role in the pathophysiology of MDD, even though it is still not completely understood [140].

The risk of depression is influenced by both genetic factors and adverse life events, particularly adverse childhood experiences (ACEs) such as childhood sexual abuse or neglect; for example, childhood sexual abuse has been associated with increased DNA methylation of the NR3C1 gene, which encodes the glucocorticoid receptor, affecting stress response pathways [141]. Epigenetic mechanisms like this can alter gene expression without changing the underlying DNA sequence, through processes like DNA methylation, chromatin remodeling, histone modifications, RNA modifications, and regulation by non-coding RNAs (ncRNAs) [142]. These changes can occur at transcriptional, post-transcriptional, translational, or post-translational levels, ultimately influencing vulnerability to depression [143].

Currently, the first-line pharmacological therapy for MDD treatment is selective serotonin reuptake inhibitors (SSRIs), which are medications that improve serotonergic neurotransmission [144,145]. SSRIs block the reuptake of serotonin, which raises the amount of serotonin at synapses. Nevertheless, between half and two-thirds of patients with MDD respond to treatment with SSRIs, and it takes weeks or months for the response to manifest [146]. Thus, antidepressant research continues to focus on obtaining a better understanding of the mechanisms underlying individual differences in the clinical response to SSRIs. Due to the unique spectrum of adverse effects, pharmacokinetic profiles, and pharmacodynamic characteristics of each SSRI, it is essential to give each patient individual therapeutic attention [147]. Based on available research, finding the right antidepressant often involves a trial-and-error process, as brain chemistry varies between individuals. Subsequent treatments, however, have shown success in as few as 40% of patients who are able to find a drug that works for them by the fourth attempt [148]. Additionally, remission and response rates differ. The following three domains are currently taken into account: (1) demographic traits (such as age, race, ethnicity, and gender); (2) social traits (such as education, employment status, income, insurance, and marital status); and (3) clinical features (such as age at onset of major depressive disorder, length of the current major depressive episode, number of major depressive episodes, length of illness, course of illness [single or recurrent], major depressive disorder subtype [anxious, melancholic, and atypical features], family history of depression, concurrent general medical and axis I psychiatric disorders, symptom severity, and functional status at baseline) [146].

#### 4.1.1. Foundation of Personalized Medicine—Pharmacokinetics and Pharmacodynamics

##### Pharmacokinetics

Fluoxetine, fluvoxamine, paroxetine, sertraline, and citalopram are five selective serotonin reuptake inhibitors (SSRIs) that have similar antidepressant effectiveness and adverse effect profiles. Nevertheless, their pharmacokinetic features and half-lives vary. Their metabolism is primarily mediated by cytochrome P450 (CYP) isoenzymes. As a result, blood levels vary significantly from person to person [149,150]. When choosing an SSRI for the treatment of depression [151], one should take into account the distinct pharmacokinetic characteristics of the five SSRIs, especially their potential for drug–drug interactions.

Antidepressants are extensively absorbed after oral administration and mainly metabolized in the liver [152]. Hepatic first-pass metabolism leads to a decrease in oral bioavailability to below 90%. The pharmacokinetics of these drugs may be affected by genetic variations in the cytochrome enzyme and hepatic metabolism through the P-450 isoenzyme system [153].

##### Pharmacodynamics

Selective serotonin reuptake inhibitors (SSRIs) share a common primary mechanism—blocking the reuptake of serotonin—but they differ slightly in their activity at other receptors and in how they are metabolized. For example, paroxetine has mild anticholinergic effects, which can contribute to constipation or dry mouth, while fluoxetine is a more potent inhibitor of cytochrome P450 enzymes, leading to potential drug interactions; meanwhile, sertraline may have mild dopaminergic activity that can influence energy or motivation. These subtle differences can affect individual tolerability and minor variations in side effect profiles, although the overall adverse effects are largely similar across the class.

Pharmacodynamic differences among SSRIs include the following:

Secondary Targets

SSRIs primarily act by inhibiting the serotonin transporter (SERT), thereby increasing synaptic serotonin. However, their effects extend beyond serotonergic transmission. Increased serotonergic activity can exert a downstream influence on dopaminergic and noradrenergic neurons, particularly in the mesocorticolimbic pathways, through 5-HT receptor-mediated mechanisms (e.g., 5-HT2C-mediated inhibition of dopaminergic firing). This serotonergic modulation of dopamine and norepinephrine systems is believed to contribute to differences in clinical effects across SSRIs [154,155]. In addition, some SSRIs display affinity for other targets, such as sigma-1 receptors, which may further influence the therapeutic response, for example, in conditions like psychotic or delusional depression [156,157].

Half-Life and Metabolism

SSRIs have different half-lives, affecting how long they stay active in the body. For example, fluoxetine has a longer half-life than the other SSRIs, and its active metabolite, norfluoxetine, also has a long half-life, which can be beneficial for some patients; however, this also increases the risk of withdrawal symptoms when discontinuing the drug. The differences in half-life and metabolism can also influence the severity of withdrawal symptoms when discontinuing an SSRI.

Clinical Effects

Pharmacodynamic variations can lead to differences in the efficacy and tolerability of SSRIs, highlighting the importance of individualized treatment approaches.

Monitoring

Clinicians should monitor patients for potential side effects and drug interactions, especially when switching between SSRIs or adding other medications [158]. Patients with poor metabolizing phenotypes exhibit high plasma concentrations of citalopram, prompting FDA recommendations for a 50% dose reduction to avoid the risk of QT prolongation [159].

Pharmacokinetic–pharmacodynamic interactions

Individualized therapies should be based on the individual SSRI’s pharmacokinetic profiles, pharmacodynamic attributes (i.e., physiological and biological effects), and unique spectrum of side effects [39,153]. For example, CYP2D6 is inhibited by the following SSRIs (in order of decreasing potency): paroxetine, norfluoxetine, fluoxetine, sertraline, citalopram, and fluvoxamine. Clinical investigations with desipramine have confirmed in vitro findings that CYP2D6 inhibition by sertraline is only moderate [155,160]. No clear-cut relationship between serum concentration and clinical effectiveness has been shown so far. The differences in half-life and metabolism can also influence the severity of withdrawal symptoms when discontinuing an SSRI.

#### 4.1.2. Architecture of Personalized Medicine

##### Pharmacogenetics

Key Genes and Mechanisms:

SLC6A4 (5-HTT)

This gene encodes the serotonin transporter protein, which is the primary target of SSRIs. Variations in SLC6A4, particularly the 5-HTTLPR polymorphism in the promoter region, can affect the transporter’s activity and thus influence SSRI efficacy. For example, the “long” allele of 5-HTTLPR is often associated with increased serotonin reuptake activity and potentially better responses to SSRIs, while the “short” allele may be linked to lower reuptake activity and an increased risk of side effects [161,162,163].

CYP2D6 and CYP2C19

These genes code for cytochrome P450 enzymes, which play a crucial role in metabolizing many SSRIs. Variations in these genes can affect how quickly an individual breaks down the medication, potentially impacting its effectiveness and side effect profile. For example, individuals with certain CYP2D6 or CYP2C19 variants may be classified as “poor metabolizers,” meaning that their bodies process the drug more slowly, potentially leading to higher drug concentrations and an increased risk of side effects [164].

Research indicates that 15% to 30% of the variability in treatment response can be attributed to genetic alleles (CYP 450 system) that affect drug absorption, metabolism, transport, and mechanism of action [27,165,166].

A recent study provided CYP2D6, CYP2C19, and CYP2B6 genotype results to help clinicians in prescribing these antidepressants and described the existing data for SLC6A4 and HTR2A, which are not presently supported for clinical use in antidepressant prescription [167].

Regarding pharmacogenomics, analysis of variations in the drug pharmacokinetics and pharmacodynamics due to the influence of the CYP450 and HLA gene families across individuals and ethnic groups helps to optimize drug dosages, minimize ADRs, and enhance overall treatment outcomes [39].

Regarding pharmacotranscriptomics, as antidepressants modulate gene expression over time in key tissues through RNA, which is the immediate product of gene expression, epigenetic modifications could be incorporated to refine treatment strategies [168,169]. Few studies have investigated the transcriptional effects of SSRI in animal models [170,171]. While antidepressants increase neurotransmitter availability (serotonin, noradrenaline, and dopamine) almost immediately, their therapeutic effects often take 4 to 8 weeks to manifest, suggesting that transcriptional changes are responsible for their clinical efficacy [170,171].

##### Pharmacometabolomics

The impact of environmental and lifestyle variables such as diet, age, nutrition, sex, and the gut microbiome [172] on drug efficacy and safety can be examined through the analysis of both baseline and dynamic post-treatment metabolic profiles, which can help to predict individual drug response variations [173].

Using targeted metabolomics, Bhattacharyya et al. (2024) analyzed metabolic alterations in 163 treatment-naive people with major depressive disorder who were treated with escitalopram, duloxetine, or Cognitive Behavioral Therapy (CBT) [174]. Serum metabolites associated with the tryptophan and tyrosine pathways were measured at baseline and after 12 weeks. The only treatment that was linked to lower serotonin levels and higher levels of tryptophan metabolites from the gut, such as indole-3-propionic acid, indole-3-lactic acid, and indoxyl sulfate [174], was antidepressant therapy; CBT did not show an effect. All treatment arms saw a reduction in purine-related metabolites. According to the authors, each treatment was linked to distinct metabolite changes that implied different mechanisms underlying the response to behavioral and pharmacological interventions [174].

##### Pharmacomicrobiomics

Studies have shown that the gut microbiota is linked to differences in SSRI response. In a longitudinal study on 30 subjects with MDD and 30 healthy controls flexibly dosed with escitalopram (up to 20 mg/day), microbial diversity was significantly higher at baseline between patients with depression and controls. A follow-up group was created that consisted of patients with depression who achieved a symptomatic response to escitalopram, defined as a 50% or greater reduction (improvement) in depressive symptoms, measured using the 17-item Hamilton Depression Rating Scale (HAM-D17). Following treatment with escitalopram, the α diversity in the follow-up group was not found to significantly differ from that of the controls measured at baseline, suggesting that escitalopram is associated with favorable changes in the gut microbiota, with the caveat that microbial metabolites after escitalopram treatment in patients with depression were still different from those of controls [175,176]. Variations in microbial composition can influence the pharmacokinetics of antidepressants and antipsychotics, altering their availability and action [177].

##### Pharmacomulti-Omics

Multi-omics approaches, integrating data from genomics, epigenomics, transcriptomics, proteomics, metabolomics, and other “omics” layers, provide valuable insights into the complex mechanisms underlying the efficacy of SSRIs in treating MDD [39]. It is a powerful approach for personalizing antidepressant treatment by better predicting individual responses and optimizing drug selection and efficacy. Antidepressant response variability and adverse effects could be due to pharmacogenetics, microbiomics, and pharmacomulti-omics. Studies have shown that integrating genomic and metabolomic data can predict responses to antidepressant pharmacotherapy with improved accuracy compared to metabolomics alone [177,178,179]. Multi-omics approaches, which integrate data from genomics, epigenomics, transcriptomics, proteomics, and metabolomics, provide critical, high-resolution insights into the biological mechanisms driving selective serotonin reuptake inhibitor (SSRI) efficacy in major depressive disorder (MDD). These methods are crucial for personalizing antidepressant treatments as they allow for better prediction of individual responses, optimization of drug selection, and reductions in adverse effects by accounting for the complex variability in pharmacogenetics, microbiomics, and pharmacomulti-omics.

By providing a holistic, systems-level view, multi-omics is transforming our understanding of depression from a uniform diagnosis to a set of distinct, biologically defined subtypes, enabling more tailored and effective therapeutic interventions.

Plasma hydroxylated sphingomyelins are prominent predictors of treatment outcomes. Sphingomyelins clustered with single-nucleotide polymorphisms and metabolites provide predictions of treatment response with different classes of antidepressants in the context of neurotransmission [101].

Multi-omics approaches aim to improve response and remission, as well as to reduce relapses. Studies using pharmacogenomics and pharmacotranscriptomic approaches, focusing on genetic variants and expression levels of relevant genes to understand the pharmacokinetic and pharmacodynamic effects of psychotropic drugs, are relevant for personalized medicine in psychiatry [180]. The implications of epigenetic abnormalities in many diseases and the approval of a number of compounds that modulate specific epigenetic targets are therapeutically relevant [181]. Selective serotonin reuptake inhibitors such as sertraline are crucial in treating depression and anxiety disorders, and studies indicate their potential as chemosensitizers in cancer therapy [182].

Regarding computational science and data intergradation, more research is needed to refine the existing predictive models and integrate them into clinical practice [182]. Table 3 lists some representative studies that support research in each “omic” layer.

### 4.2. Example #2: Statins

Statins are effective in lowering low-density lipoprotein cholesterol and treating/preventing atherosclerotic disease. Lipophilic statins easily pass through cell membranes and include atorvastatin and simvastatin (both metabolized by CYP3A4), along with pitavastatin (which undergoes minimal metabolism, mainly by glucuronidation rather than the action of CYP4502C9).

Hydrophilic statins feature higher liver selectivity, with rosuvastatin barely metabolized by CYP450, and fluvastatin metabolized by CYP450 2C9 [183]. All statins, especially at high doses, are associated with high blood sugar levels. The risk of new-onset DM is 48% higher in statin users [184,185]. The only statin that has no effect on blood glucose is pitavastatin [186]. However, the beneficial effects of statin therapy outweigh its potential harms in diabetes, with a NNH (Number Needed to Harm) of 498 versus a NNT (Number Needed to Treat) of 155 [187]. Engeda et al. performed a meta-analysis of eight randomized controlled trials and 15 observational studies and found an association between statin use and new-onset diabetes mellitus, showing that the risk was higher in observational studies (relative risk: 1.55; 95% CI: 1.39–1.74) than in randomized controlled trials (relative risk: 1.11; 95% CI: 1.00–1.22) [188].

The concept of multitarget pharmacology—also known as pleiotropic effects—is particularly intriguing in the case of statins. Different statins can have variable effects and tolerances due to their off-target effects, which can vary significantly from one individual to another. Switching to a different drug within the same class may result in different off-target effects, which may be beneficial or detrimental [189]. A considerable proportion of people reduce their dose or discontinue treatment. Currently, healthcare providers prescribe statins based on few data elements, with the best disease prevention and treatment plans considering individual genetic backgrounds, environments, and lifestyles. The transport and metabolism of statins are complex processes involving numerous enzymes and transporters, making it challenging to regulate them [190]. Guidelines suggest selecting treatments based on LDL cholesterol levels, but not based on patient profiles, which may lead to side effects like muscle pain or diabetes.

#### 4.2.1. Foundation of Personalized Medicine—Pharmacokinetics and Pharmacodynamics with Statins

The genetic expression of genes involved in the ADME of statins results in considerable interindividual variation in their pharmacokinetics and pharmacodynamics, which cannot be fully accounted for by genetic polymorphisms. Epigenetic factors, such as DNA methylation, histone modifications, and transcriptional regulation mediated by non-coding RNAs (ncRNAs), also have important regulatory functions. Epigenetic regulation governs a large number of genes that code for metabolic enzymes, transcription factors, transport proteins, and drug targets, all of which have significant effects on the pharmacokinetics and pharmacodynamics of statins [191,192,193].

#### 4.2.2. Architecture of Personalized Medicine with Statins

##### Pharmacogenetics

Genetic variations such as SLCO1B1, ABCG2, and CYP2C9 influence the effectiveness of statins, as well as the incidence of side effects like myopathy [194].

Genetics alone do not explain all the variance in drug response, with epigenetic modifications such as DNA methylation (DNAm) having also been implicated in this regard [35,195]. These epigenetic changes are not only responsible for the variability in drug response, but also contribute to the pleiotropic benefits and adverse effects (e.g., altered DNA methylation at ABCG1 linked to increased diabetes risk). A comprehensive understanding of the related epigenetics is crucial for achieving personalized statin therapy.

##### Pharmacomicrobiomics

New evidence has shown that the gut microbiome modulates the efficacy and side effects of statin therapy through alterations in the drug metabolism and therapeutic response [196], and gut microecology mediates the therapeutic effects and adverse reactions of statins. Thus, the gut microbiota, as a complex ecosystem of microorganisms within the gastrointestinal tract, plays a critical role in influencing the therapeutic and side effects of statins [197]. An association has been found between a certain microbiome composition and the lipid-lowering effect of statins [198]. Certain phyla in the gut microbiome, like Firmicutes and Bacteroides, cause metabolic disruption and altered statin responses [179]. Simvastatin–bile acid–microbiome interactions are also important, and the bioaccumulation and biotransformation of simvastatin by intestinal bacteria might be the mechanisms underlying altered simvastatin bioavailability and therapeutic effects [199].

##### Pharmacomulti-Omics

Studying multiple transcription factors and epigenetic regulators is necessary to identify targets for accurate statin treatment [192]. The same treatment can have varying responses in different subjects [200] due to genetic polymorphisms, which can be studied via pharmacogenetics and pharmacogenomics as well as through the transcriptional regulation of metabolism. The transport and metabolism of statins involve numerous enzymes and transporters, making it challenging to regulate them effectively by targeting a single enzyme or transporter [192]. Multi-omics helps to reduce the reliance on trial-and-error approaches for statin therapy. Genetics may partly explain adverse drug reactions (ADRs). In addition, Smith D et al. found 17 DNA methylation (DNAm) sites (epigenetics) altered by statins, which may explain ADRs [201].

Regarding computational science and data integration, using data integration techniques, Liu et al. found that the estimates of statins’ effects on metabolites in cross-sectional studies were comparable to those from intervention and genetic observational studies [202]. Specific multi-omics studies with large sample sizes are summarized in Table 2.

### 4.3. Investigations into Personalized Medicine

#### 4.3.1. Testing/Markers for Pharmacomulti-Omics

In a realistic cohort of depressed patients and healthy controls [39], three genome-wide sequencing and microarray techniques were used to create expression profiles for protein-coding genes and microRNAs, as well as to analyze DNA methylation levels in peripheral blood samples. Investigations are also pushing towards multi-omics approaches, which aim to target specific protein forms and metabolites, rather than just genes, promising even higher precision in drug therapy.

Systems biology integrates transcriptomics, proteomics, and metabolomics with clinical data to understand the holistic impact of depression at the cell, tissue, and organ levels. These comprehensive investigations aim to move psychiatry from a trial-and-error approach to a tailored, data-driven methodology.

Regarding pharmacogenomics (PGx) integration, research typically focuses on identifying genetic variants—such as cytochrome P450 enzymes (CYP450) or TPMT—that dictate drug metabolism; for instance, PGx testing helps clinicians to determine appropriate doses for medications like warfarin to prevent bleeding risks.

#### 4.3.2. Metabolomics

Metabolites—the products of cellular processes—can also be used as biomarkers. For example, specific metabolites have been associated with remission in patients treated with SSRIs [203].

Systems biology describes changes in biology associated with transcriptomics, proteomics, and metabolomics, as well as physiological changes triggered by gene expression at the cell, tissue, organ, and body levels. Both biological and physiological perturbations can be evaluated using biomarkers (e.g., peptides, deoxyribonucleic acid (DNA), ribonucleic acid (RNA) or imaging parameters) that apply to areas of clinical disease management.

Regarding AI-driven personalized prescription, new methods have integrated drug–drug (DDI), drug–gene (DGI), and drug–lifestyle (DSI) interactions into AI models (e.g., KM2PS) to generate personalized, safer prescriptions.

## 5. Proposed Integration at Different Pharmacological Levels in the New Personalized Medicine Model (Figure 3)

### 5.1. Integration of Different Mechanisms/Pathways for the New Personalized Medicine Model

- Oral cavity: Intake—pharmacomicrobiomics and pharmacokinetics.
- Gut and liver: Cycle between pharmacomicrobiomics and pharmacoecology, and then pharmacokinetics and pharmacodynamics using multi-omics approaches.

The gut microbiota can directly transform drugs through enzymatic activity, influence host enzymes in the liver and gut, modulate the immune system, and alter drug absorption through changes in pH and other factors, creating a continuous cycle of drug metabolism and host–microbe interactions that is crucial for understanding personalized medicine. The gut microbiome can influence host factors, such as the expression of liver enzymes or the production of metabolites, like short-chain fatty acids (SCFAs), which can alter drug pharmacokinetics.

By initiating oxidative stress, immune responses, and inflammatory processes, drugs can cause hepatic damage either directly (through their intrinsic hepatotoxic effects) or indirectly, which may result in the death of hepatocytes. The significance of the gut microecology in human health and illness is widely acknowledged, with recent research having demonstrated that an imbalance in the gut microecology is strongly associated with the etiology of drug-induced liver injury (DILI) and plays a significant role in the disease course [204].

c. Whole-body system: Cellular mechanisms play a main role through multi-omics pharmacology.
  1. Pharmacometabolomics and multi-omics pharmacology.
  2. Cellular mechanisms: Both pharmacodynamics (receptor- and signaling pathway-related) and multi-omics, including genomics, focus on the entire genome (all of an organism’s genes) and their interactions, as well as the genetics (pharmacogenetics and pharmacogenomics) of both humans and microbiomes. Omics alterations (epigenetic, transcriptomic, proteomic, and metabolomic alterations as a consequence of drug exposure) can help clinicians to ascertain patients’ cellular responses to medications. The proposed model suggests that a “one-size-fits-all” dosage fails because individual microbiome signatures differ. By analyzing a patient’s microbiome (e.g., assessing the abundance of drug-metabolizing bacteria), clinicians can predict how a drug will behave, leading to safer, more effective personalized treatment regimens.
d. Kidneys: Elimination pharmacokinetics.

The integration process in the new personalized model is as follows. Most of the medications for the treatment of various diseases are available as oral medications (tablets/liquids). Once an individual consumes the medication, the microbes in the oral cavity interact with it through biotransformation (e.g., by metabolizing it) as well as through altering its pharmacokinetics, which can influence the absorption of the medication and its bioavailability. Additionally, medications can cause a change in the oral microbiome. Once the medication reaches the gut, it is subjected to metabolism by the gut microbiome and liver, as well as the first-pass pharmacokinetic effect due to the liver and pharmacodynamics. Pharmacodynamic and pharmacokinetic processes are influenced by the host and microbial genetics and various other host cell mechanisms, such as signal transduction and cellular/receptor communication. Eventually, the drug is typically converted into various non-active metabolized end products through the liver and through pharmacokinetic processes in the kidneys, and finally eliminated from the body.

As previously mentioned, pharmacogenomics is essential as it aims to assess and validate the impact of human genetic variation on drug responses [26,27]. Moreover, metabolomics is a crucial approach in understanding disease pathogenesis and helps to provide better diagnoses and treatments. The development of ‘omics’, which integrates data from genomics, transcriptomics, proteomics, metabolomics, and other “omics” fields into a personalized medicine approach, involves integrating pharmacological principles such as pharmacokinetics and pharmacodynamics with pharmacomicrobiomics to create a multi-omics profile. This integration will potentially help healthcare professionals develop personalized treatment strategies.

Diseases in humans affect multiple organs/systems and change metabolite and protein concentrations in the body. Analysis of affected metabolites or proteins provides an effective means to monitor drug treatment interventions in terms of efficacy and side effects [205]. Nuclear medicine resonance (NMR) spectroscopy and mass spectrometry (MS) are the most common and powerful analytical tools for studying metabolic markers [206,207], genes, and single-nucleotide polymorphism (SNPs) (genomics), small, biologically active molecules (proteomics and metabolomics), and even the metabolic pathways of individuals (metabolomics) [208,209]. In addition, personalized medicine takes into account not only the physiological/pathological status of a person’s body, but also the unique psychosocial situation of the individual, which may have direct effects on when a given health condition manifests in that individual and how they respond to treatment [210]. Currently, it is known that inherited variations in approximately 20 genes can affect around 80 medications and the way the body responds to them [20,211]. Overall, using multi-omics will help researchers to identify biomarkers for the prediction of drug sensitivity or resistance as well as the development of personalized treatment plans for individuals. Multi-omics can also help to predict drug responses based on individual molecular profiles, leading to more personalized treatment plans.

**Figure 3:**
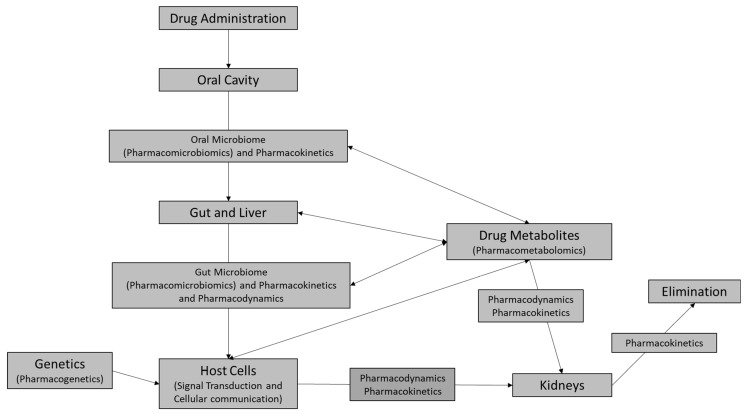
New model of the various components and mechanisms of the pharmacological process. When a drug is administered into the oral cavity, it begins interacting with the host via pharmacokinetics and pharmacomicrobiomics (oral microbiome) pathways. As it is processed through the gastrointestinal tract, the drug is again exposed to pharmacokinetics and pharmacomicrobiomics mechanisms. Through the oral cavity, gut, and liver, some substances can be broken down into specific metabolites that can have bioactive properties on the host (and even gut microbiome) [Pharmacodynamics]. The drug metabolites can interact with host cells through various communication and signal transduction methods. The host’s genetics/genome can also play a role in how the host cells respond to these metabolites (pharmacogenetics). As the drug is processed by the host, it is eventually eliminated by the body through the kidneys, where some pharmacodynamic and pharmacokinetic influences may persist. This more multi-omics approach can help to build a model that is oriented towards more personalized medicine.

### 5.2. Implementation Framework

**Figure 4:**
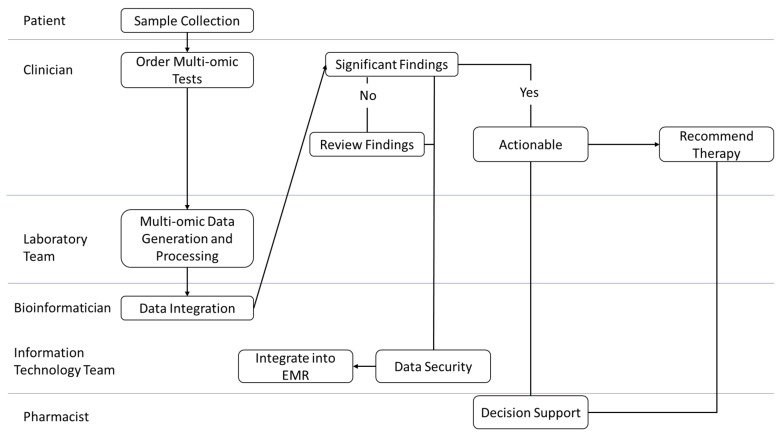
Workflow diagram for multi-omics clinical testing and integration into clinical practice. This figure illustrates a presumed end-to-end workflow for implementing multi-omics testing into clinical practice. The initial step involves collecting patient samples followed by multi-omics testing, which are ordered by the clinician. Collected samples are processed in the laboratory and the multi-omics data are generated and processed. Then, a bioinformatician conducts the data processing analysis and integration. The generated results are evaluated for possible clinical significance. If none are detected, the results are then documented into the patient’s electronic medical record (EMR) without further action. If significant results are obtained, the information is reviewed and communicated with the clinical team/ordering provider. Again, the results are entered into the patient’s EMR with the support of the information technology team, ensuring compliance with data security requirements. The findings obtained can help to guide personalized therapeutic recommendations with the support of a pharmacist to further guide clinical decisions alongside the care team.

The implementation of this model involves different stakeholders, such as information technology (IT) professionals, and also a need for clear decision support protocols. A workflow diagram highlighting this framework is shown in Figure 4, which captures multi-omics data, analysis, and translation as actionable recommendations. Specific multi-omics studies with large sample sizes are summarized in Table 2.

The main point is that a thorough understanding of the practical and regulatory obstacles that might impede clinical or commercial applications is necessary for effective, real-world translation, along with a basic grasp of the scientific possibilities of omics data. Replication is essential for any scientific conclusion to be deemed trustworthy. Before relying on any particular omics technology for translation, readers are advised to carefully consider how consistently its findings can be reproduced across various laboratories, platforms, and patient populations.

Regarding regulatory hurdles, the transition of research from laboratory to the bedside frequently necessitates navigating complicated regulatory environments (e.g., FDA or EMA approval) [212].

To integrate a new individual-level model with a population health approach and increase its impact on the health system, various methods should be included. Equity stratification is performed to disaggregate health indicators and outcomes, using key demographic, social, economic, racial, or geographic characteristics, in order to identify and measure differences across population subgroups.

Macro-level public health data, such as community-sourced social determinants of health (SDOH) data, are used to construct a more complete picture for analysis within the cost-effectiveness framework [213], thus incorporating health equity issues.

**Figure 5:**
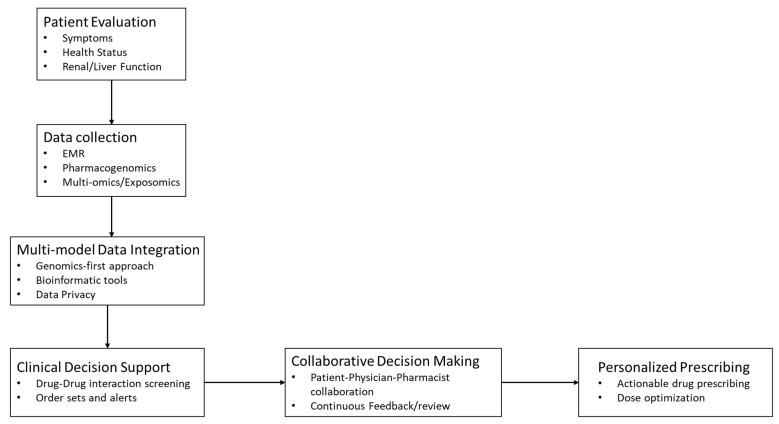
Clinical decision-making framework for personalized medicine medication management. This workflow distinguishes steps that are feasible today from those representing next stage multi-omics expansion while clarifying the primary responsibility for each step. Current capabilities include integration of pharmacogenomics (e.g., CYP2D6/CYP2C19), drug–drug interaction screening, laboratory and organ function data, problem lists, and patient goals into EHR-embedded CDS tools, supported by pharmacist review and clinician decision making. Tools such as PharmCAT (for genotype extraction and annotation, including CYP2D6 diplotypes) exemplify current bioinformatic integration. In contrast, future components—including proteomics, metabolomics, and microbiome data streams—require additional laboratory workflows, bioinformatic processing, and validation before routine deployment. The figure also highlights role ownership: clinicians initiate testing and incorporate results into shared decision making; laboratories/bioinformatics generate and process PGx and multi-omic data; IT/EHR teams manage data harmonization and CDS integration; and pharmacists perform medication optimization and monitoring planning.

A practical clinical workflow with the implementation framework is shown in Figure 5. Implementing a personalized medicine data infrastructure requires a systematic approach to bridge the gap between complex biological data and everyday clinical practice, aggregating data from electronic health records (EHRs) on genetics (pharmacogenomics), multi-omics, liver/renal function, and social/lifestyle factors. The specific steps of the workflow are as follows. 1. Create databases and data warehouses with an open-science model. 2. Develop user-friendly, accessible bioinformatic tools for data mining and discovery. 3. Employ a genomics-first approach to subtype complex diseases and develop accurate biomarkers and targeted therapeutics. Integrated multi-omics is used to compose the symphony of biological data. Multimodal data are collected, combining clinical, genomic, and multi-omics data like transcriptomic, proteomic, and metabolomic data. Then, multimodal data software is used to obtain a more holistic view of the disease, access a different layer of biological systems, and realize medication management, as well as address data privacy and regulatory concerns. In this manner, clinicians can overcome the complexity of data integration and interoperability.

The implementation framework should involve practical clinical workflows enabling the capture and analysis of multi-omics data, as well as the translation from decision support protocols to actionable drug recommendations, with supporting IT requirements and clear stakeholder roles. A clinical decision-making framework for personalized medicine with medication management involves the following steps and processes, as summarized in Figure 5.

Data Collection and Integration: Data on genetics (pharmacogenomics), multi-omics, liver/renal function, and social/lifestyle factors are first aggregated from electronic health records (EHRs). Then, databases and data warehouses are created with an open-science model; user-friendly, accessible bioinformatic tools are developed for data mining and discovery; and a genomics-first approach is designed to subtype complex diseases and develop accurate biomarkers and targeted therapeutics. Integrated multi-omics is used to compose the symphony of biological data.

Multimodal data combining clinical, genomic, and multi-omics (transcriptomics, proteomics, and metabolomics) data should be collected, and multimodal data software should be developed to obtain a more holistic view of diseases and assess different layers of biological systems for correct medication management, as well as to address data privacy and regulatory concerns. In this manner, clinicians can overcome the complexity inherent to data integration and interoperability.

The Pharmacogenomics Clinical Annotation Tool (PharmCAT) is an example of a genomics data collection tool that can be integrated into the following processes.

Interaction Screening: Automatic screening for drug–drug, drug–gene, and drug–herb interactions can be beneficial for clinicians.

Intervention: Pop-up alerts, dynamic order sets, and documentation assistants can be useful.

Collaborative Decision Making: The patient is involved in defining goals, monitoring symptoms, and adjusting regimens [16].

## 6. Future Studies to Validate New Personalized Medicine Model for Medication Management

To demonstrate that the new Personalized Medicine Model for medication management improves patient results compared to traditional approaches, future validation studies should follow a multi-phased methodology that includes technical feasibility, pilot studies, follow-up cohorts and, ultimately, randomized controlled trials (RCTs). The first step involves focusing on internal validation utilizing retrospective diverse datasets to verify the model’s accuracy and dependability in a regulated environment, emphasizing its technical feasibility. The following step involves prospective pilot studies to evaluate the model’s accuracy and safety in preparation for more extensive trials and to collect early proof of effectiveness. Then, a randomized controlled trial (RCT) should be carried out to compare the model with current approaches and analyze various clinical and technical indicators of effectiveness and safety. Finally, post-market surveillance should be conducted to assess its real-word performance and uncover potential safety hazards.

AI-driven healthcare solutions can help to integrate a wide variety of data types and facilitate individualized treatment planning, and offer enhanced healthcare services [214]. Machine learning models can be assessed through metrics such as confusion matrices, accuracy, sensitivity, specificity, and F1 score, as well as ROC curves, to understand their strengths and weaknesses [215].

Multiple data sources, such as electronic health records (EHRs), laboratory results, omics data (genomics, epigenomics, proteomics, transcriptomics), and imaging data, should be integrated via multimodal data integration in order to offer thorough, individualized insights.

To support clinical utility beyond theoretical benefit using machine learning, the model must be assessed quantitatively, such as with regard to decreases in adverse drug reactions (ADRs) and improvements in efficacy. Although the field is moving closer to producing the kind of quantitative, outcome-based evidence clinicians are looking for, large-scale, reliable findings on direct patient outcomes, such specific percentage reductions in ADRs, are still emerging as research moves from algorithmic potential to clinical implementation, mainly in the context of cancer treatment [216].

## 7. Ethical and Regulatory Concerns

### 7.1. Regulatory Concerns

As omics research is complicated, regulatory bodies such as the US Food and Drug Administration (FDA) and European Medicines Agency (EMA) have been hesitant to adopt the associated data. They have also been careful to ensure that the tests used in omics-based tests are adequately validated for use with novel measurement platforms and that they exhibit high statistical validity in differentiating patient subgroups. Regulatory hurdles include creating standards for the kinds of omics data that should be gathered to thoroughly validate the full procedure using well-defined regulatory routes.

In vitro assays and in vivo animal studies are typically used as the basis for initial testing. To mimic intricate human physiology, multi-organ micro-physiological systems are being developed as it is crucial to simulate how several organs and systems interact. Nevertheless, the pharmaceutical industry is attempting to lessen its reliance on conventional animal models, particularly when creating novel treatments that target genes or pathways unique to humans and not present in animals, which promotes the development of testing methods that are more relevant to humans [217].

Several authors have emphasized the significance of algorithmic auditing and well-defined liability frameworks to ensure accountability throughout the AI lifecycle [202,203] as regulators like the FDA and European Medicines Agency (EMA) are developing AI-specific guidance for clinical use. Other recommendations include creating dynamic consent models that allow patients to update their data-sharing preferences over time [218] and establishing uniform regulatory standards to promote the safe translation of AI-based medical devices from research to practice [219].

### 7.2. Replacing Animal Models

To better mimic human physiology, the pharmaceutical industry is developing multi-organ microphysiological systems (MPSs), also known as “organs on chips”. These systems allow for more relevant drug testing, particularly with respect to pathways that are unique to humans, with the aim of reducing reliance on conventional animal models.

MPSs are designed to resemble the 3D structure, cell types, and extracellular matrix of human organs. A key advantage of an MPS is its high customizability, enabling researchers to create models that simulate specific organs, physiological conditions, or diseases [220]. Both the US Food and Drug Administration (FDA) and European Medicines Agency (EMA) are actively developing frameworks for these technologies, aiming to foster the safe translation of advanced, high-dimensional data into clinical practice.

### 7.3. Ethical Concerns

The possibility of traditional privacy and consent issues is increased when using multi-omics data (such as genomic, proteomic, and imaging data). In many multi-omics studies, where data uses might change over time, the traditional, one-time “specific consent” model is frequently insufficient. Because multi-omics is scientifically complicated, patients may have trouble comprehending the consequences of data sharing, which might result in inadequate informed consent. In the end, a moral framework for multi-omics in personalized medicine must strike a balance between data accessibility for scientific progress and the fundamental values of patient autonomy, privacy, and justice [221,222].

Although many researchers have emphasized uncertainty around the use of secondary data and the sufficiency of current consent models when patient data are continually repurposed for the development of AI [218], little research has been conducted on the deployment of AI in precision medicine, much less with a focus on technical, ethical, and workflow considerations [223].

## 8. Discussion and Conclusions

With a better grasp of multi-omics–host dynamics, the proposed model may offer insight into novel therapeutic approaches; however, further clinical evaluation tools are necessary.

According to current estimates, between 20% and 95% of the variability in response to specific medications may be attributed to genetic causes [28]. The missing piece in drug efficacy could be the use of pharmacomicrobiomics and pharmacoecology as we know that the human gut microbiota plays a key role in metabolism. Genetics, age, gender, lifestyle, illness status, drug–drug interactions, environmental factors, and the gut microbiota all contribute to IVDR, which is only partially explained by a relatively small number of coding genes in the human genome. The gut microbiota, often referred to as the second genome, influences the effectiveness of treatments for human illnesses and may impact drug PK and PD due to alterations in pathological conditions, metabolic enzyme expression, and drug transporter expression. It is essential to comprehend these variables in order to maximize pharmacological treatment and reduce the chance of adverse drug reactions (ADRs).

Overall, when optimized, personalized medicine is expected to improve first treatment choices and avoid unnecessary medication and reduced trial-and-error drug management, as well as improve patient safety by reducing the incidence of adverse drug reactions and reducing overall healthcare utilization.

Traditionally, medical/psychiatric diagnoses rely on signs and symptoms categorized under broad syndromic labels, with treatments often guided by a trial-and-error approach. This empirical method exposes patients to unnecessary risks and delays in achieving optimal outcomes [224]. Optimizing drug therapy is an essential part of patient care. While determining an appropriate dose and schedule for the patient’s physiologic status (pharmacokinetics and pharmacodynamics) is important, monitoring for effectiveness and toxicity is also essential (in this regard, personalized medicine may prove helpful). Current practices following clinical practice guidelines target the population level, but may not work as intended at the individual level due to patient-specific variations resulting from pharmacokinetic and pharmacodynamic responses, in addition to other factors. Using the right drug, at the right dosage, for the right patient, and at the right time is important. Recent emerging evidence points out that considering the whole-body microbiome as an invisible new organ through pharmacomicrobiomics may also play a role at the individual level. Microbiome-based therapeutics have the potential to improve medication management at an individual level. Pharmacomicrobiomics will undoubtedly play a crucial role in shaping the future of all medical treatments. Gut microbiota modulation via probiotics, prebiotics, synbiotics, fecal microbiota transplantation (FMT), or personalized nutrition may also prove to be a potential strategy for preventing an adverse reaction or improving the drug response when treating different diseases.

Pharmacokinetics, pharmacodynamics, pharmacogenomics, pharmacomicrobiomics, pharmacomulti-omics, and pharmacoecology should all be considered in treatment decisions. Clinicians should assess the drug’s safety and effectiveness in treating a recognized illness. Placing high value on individual variability as a key component, PM has become a cutting-edge method for managing illnesses. At present, PM concentrates on molecular and biochemical targets in a variety of medical diseases. Furthermore, a top-down strategy (from the oral cavity to the kidneys) can reveal additional pathophysiological factors that explain how medicines work and how diseases develop. PM makes use of the synergistic integration of numerous fields—such as genetics, neurobiology, pharmacology, and computational biology—to create a more logical individualized treatment for medical disorders. Omics approaches are particularly important among the available tools for comprehending neurometabolic mechanisms [225]. Over time, metabolomic screening has improved our knowledge of the dynamics of disease, demonstrating that metabolite levels are impacted by both genomic (unmodifiable) and environmental (modifiable) variables [226].

Pharmacogenetics can be used to evaluate the static view of drug responses, while transcriptomics, proteomics, and metabolomics can be used to assess the dynamic view. Computational biology can be used to create multidimensional representations of the complete hierarchical system of medication responses in healthy, sick, and treated individuals. Utilizing proteomics and metabolomics, we can employ mass spectrometry to find and measure proteins, metabolites, and lipids, as well as to determine the shifts that occur during illness or after treatment. With advances in the use of biomarkers and instruments for multi-omics analysis, drug–drug interactions can be better understood through a tailored strategy in terms of not only shared metabolic routes, but also drug–microbiome and drug–metabolite interactions.

The direct metabolism of drugs via gut microbial activity, modulation of the expression of relevant genes, and competitive inhibition utilizing their metabolites [227] are the primary mechanisms presented in this review. The most promising and clinically feasible methods for managing pharmacomicrobiomic interactions include the co-administration of gut-active supplements (such as pro- and pre-biotics) and novel drug delivery methods. By precisely mediating PK/PD while reducing metabolic problems caused by drug-induced gut dysbiosis [228], these approaches that target the gut microbiome provide novel avenues for increasing therapeutic efficacy.

Regarding data standards, the lack of uniform or consistent taxonomies, formats, and data entry processes across different systems and institutions hinders interoperability and data quality. Significant financial investment is required for initial start-up, ongoing maintenance, necessary infrastructure, and personnel training [229].

Healthcare providers must prioritize data privacy and security, which may be achieved through the use of sophisticated encryption methods, enforcing stringent access restrictions, and following all applicable regulatory frameworks, such as the Health Insurance Portability and Accountability Act (HIPAA) [230].

This review discusses and provides an innovative holistic view of personalized medication treatment strategies; however, more research is needed to translate this information model into clinical practice.

By allowing for more precise predictions of drug responses and the discovery of new therapeutic targets, network-based multi-omics integration offers a potent framework for drug discovery. Studies have demonstrated the complementary advantages of various methodological approaches, ranging from network propagation and similarity-based techniques to sophisticated graph neural networks and network inference models. Although there has been great progress in enhancing biological interpretability and prediction accuracy, there are still significant issues in dealing with data heterogeneity, computational scalability, and the standardization of assessment frameworks [231,232]. The integration of new single-cell and spatial omics technologies presents both prospects and obstacles, necessitating the development of innovative computational approaches that can handle the increased complexity of the data while maintaining their biological significance [233]. Additionally, the industry has to address the trade-off between model complexity and interpretability, notably in the context of cutting-edge machine learning techniques. Future advances should focus on a few important areas, including the integration of temporal and spatial dynamics into network models, the improvement of model interpretability without compromising predictive accuracy, the creation of scalable algorithms for large multi-omics datasets, and the development of uniform evaluation frameworks. Successfully overcoming these challenges will be essential in turning computational findings into practical clinical uses, which is expected to ultimately advance the domains of precision medicine and drug development.

Future studies should focus on integrating various types of data, creating sophisticated computational tools, and incorporating AI and machine learning to promote personalized medicine applications [234], as well as developing models that are predictive, adaptable, and interpretable in various medical settings, possibly involving the integration of multi-omics data, longitudinal patient records, and environmental factors to create comprehensive integrated models. Integrating omics research with real-world, multimodal medical data is critical for general advancement in clinical practice [235].

By discussing personalized medicine holistically, this article illustrates its broader implications. With two case examples, it presents a cohesive narrative of how personalized medicine can be responsibly and sustainably integrated into healthcare. This article also outlines directions for future work.

## 9. Future Directions

Further work that considers how the value of personalized medicine varies with different stakeholders, including healthcare professionals, patients, and hospital facilities, is needed. From the perspective of the patient, the value obtained from a personalized solution is based on the increase in quality of life due to the avoidance of side effects, better outcomes, and how the new solution reduces out-of-pocket expenses. For healthcare professionals, value arises from the improvements in clinical outcomes and how it reduces their workload, while hospitals are generally more interested in how a personalized solution reduces the patient’s length of stay, the average unit costs for treating each patient, and the effect the treatment has on the organization’s key performance indicators. Future studies should focus on these outcomes. Future research in personalized medicine medication models should focus on integrating multi-omics data (genomics, proteomics, and metabolomics) with AI-driven, real-world data for predictive, precise drug selection.

## Figures and Tables

**Table 2:** The pharmacological process: definitions and processes.

Step	Definition	Process	Reference
Pharmacokinetics (PK)	The study of the interaction and influence of the host body on administered substances/drugs/medications throughout the duration of exposure.	Examines the overall time course of a specific drug’s concentration in blood/tissue, as well as how specific drugs are absorbed, distributed, metabolized, and excreted from the body.	Grogan and Press. (2023) [33]
Pharmacodynamics (PD)	The study of the effects that substances/drugs/medications have on the host body.	Describes the process in which substances/drugs/medications interact with cellular receptors, enzymes, or other targets to exert their therapeutic or toxic effects.	Marino et al. (2023) [34]
Pharmacogenetics	The study of how genetic variations influence an individual’s response to drugs.	Focuses on understanding genetic markers that affect drug metabolism, efficacy, and toxicity, leading to personalized medicine.	Smith et al. (2023) [35]
Pharmacoepigenetics	The study of how epigenetic modifications can lead to variation in an individual’s response to a medication/drug.	Focuses on identifying variations in epigenetic markers, selecting key epigenetic biomarker(s), and then mapping these biomarker(s) to a drug response phenotype. From this, compounds can be used to target epigenetic mechanisms to alter gene expression.	Smith et al. (2023) [35]
Pharmacomicrobiomics	The study of how the microbiome (microorganisms living in the body) affects the pharmacological response to drugs.	Investigates how the gut microbiota modulates drug metabolism, efficacy, and toxicity.	Rizkallah et al. (2010) [36]
Pharmacoecology	The study of how drugs impact the environment, particularly ecological systems.	Focuses on the environmental consequences of pharmaceutical use and its impact on ecosystems, wildlife, and plant life.	Flexner (2008) [37]
Pharmacometabolomics	The study of metabolic changes and the metabolites produced as a result of drug administration.	Uses metabolic profiling to assess how drug treatments influence metabolic pathways and biomarkers.	Beger et al. (2020) [38]
Pharmacomulti-omics	A comprehensive approach for studying the interactions of different biological omics (genomics, proteomics, metabolomics, etc.) in the context of pharmacology.	Combines data from genomics, transcriptomics, proteomics, and metabolomics to fully understand drug action on a systems level.	Dhieb and Bastaki (2025) [39]
Organ/System Mechanisms	Mechanisms related to how the drug interacts with the body at the organ/system level.	Describes how the body absorbs, distributes, metabolizes, and eliminates the drug (PK) and how the drug affects the body (PD).	Zhao et al. (2012) [40]
Molecular and Cellular Mechanisms	Mechanisms at the molecular and cellular levels, including how drugs influence signal transduction and cellular communication.	Covers the biochemical pathways and cellular responses that mediate the drug’s effects.	Su et al. (2024) [41]
Cell Signaling	The process by which cells communicate with each other through signaling molecules to regulate cellular activities.	Involves receptors, second messengers, and other molecules that mediate the drug’s action on a cellular level.	Su et al. (2024) [41]

**Table 4:** Comparison of conventional medicine model versus multi-omics Personalized Medicine Model.

Feature	Conventional Model	Personalized Model
Primary Data Inputs	Clinical Symptoms Patient demographics Basic labs Physical exam	Genomics Transcriptomics Proteomics Metabolomics Microbiomics
Understanding of Drug Response	Population averages/reports/studies	Individualized
Role of the Microbiome	Not included	Integrated into drug metabolism and efficacy
Decision Support	Clinician judgment Guideline-based	Algorithmic Physiologically based pharmacokinetic modeling Predictive machine learning
Evidence Model	Observation Randomized control trials	Discovery leading to validation, and then modeling and finally trials

## Data Availability

Not available.
